# Employment status and depressive symptoms in taiwanese older adults: an 11-year prospective cohort study

**DOI:** 10.1186/s12877-024-05258-w

**Published:** 2024-08-09

**Authors:** Ping Shih, Ming-Yu Lin, Yue Leon Guo

**Affiliations:** 1 Department of Environmental and Occupational Medicine, National Taiwan University (NTU) College of Medicine and NTU Hospital, Rm 339, 3F., No. 17, Xuzhou Rd., Zhongzheng Dist, Taipei City, 10055 Taiwan; 2 Department of Environmental and Occupational Medicine, National Taiwan University Hospital Hsin-Chu Branch, Hsinchu, Taiwan; 3 Department of Industrial Education, National Taiwan Normal University, Taipei, Taiwan; 4 Ministry of Education, Taipei, Taiwan; 5 Institute of Environmental and Occupational Health Sciences, College of Public Health, National Taiwan University, Taipei, Taiwan

**Keywords:** Depressive symptom, Older adult, Mental health, Repeated measures, Working status

## Abstract

**Background:**

Taiwan became an aged society in March 2018, and it is expected to become a super-aged society by 2025. The trend of increasing proportions of older adults continuing to work is inevitable. However, few studies have been conducted to investigate the effects of employment on the mental health of older adults. Therefore, we longitudinally explored the relationship between employment status and depressive symptoms in Taiwanese older adults.

**Methods:**

The study included 5,131 individuals aged 50 and above, of which 55.6% were men, who had participated in the national-wide Taiwan Longitudinal Study of Aging in 1996, 1999, 2003, and 2007. Of them, 1,091 older adults had completed all four surveys. Depressive symptoms were assessed using the Center for Epidemiological Studies of Depression scale; the total score on this scale ranges from 0 to 30. Employment status was assessed during each survey wave. Logistic regression was performed using a cross-sectional design. The effects of unemployment on depressive symptoms were analyzed using a generalized estimating equation model with a repeated measures design.

**Results:**

In each survey wave, employed older adults exhibited better mental health than did unemployed ones. After adjustments for potential confounders, unemployment was found to exert a significant adverse effect on depressive symptoms. The repeated measures analysis revealed that employment protected against depressive symptoms, as noted in the subsequent surveys conducted after 3 to 4 years (aOR [95% CI] = 0.679 [0.465–0.989]).

**Conclusion:**

Employment may reduce the risk of depressive symptoms in community-dwelling older adults in Taiwan.

**Supplementary Information:**

The online version contains supplementary material available at 10.1186/s12877-024-05258-w.

## Introduction

In 1993, Taiwan became an aging society, with approximately 7% of its population being aged ≥ 65 years. However, in March 2018, this proportion increased to 10% and Taiwan became an aged society. Taiwan is expected to become a super-aged society by 2025, with approximately 20% of its population being aged ≥ 65 years. Population aging in addition to a reduced fertility rate altered the country’s demographic structure; these changes include a gradual decline in the total number of working-age individuals (people aged between 15 and 64 years) from its peak in 2015. In Taiwan, the rate of labor force participation is approximately 59%; this rate is high at 90% among individuals aged 25–39 years. This age group has been constituting the main working-age labor force since 2019. However, the labor force participation rate has substantially declined among individuals aged > 50 years. In 2019, the rates of labor force participation were 85%, 74%, 56%, 37%, and 8.32% among individuals aged 45–49, 50–54, 55–59, 60–64, and ≥ 65 years, respectively. To boost labor participation among older job seekers, the Taiwanese government enacted the Middle-aged and Elderly Employment Promotion Act—a special, standalone act that establishes the rights of employed older adults—in December 2019. The Act is primarily enacted to promote decent work and increase labor participation among individuals aged 45 and older. It aims to facilitate the re-employment of older adults, ensure economic security, foster intergenerational cooperation and experience sharing, protect employment rights, create a supportive work environment, and enhance the utilization of human resources [1].

Conditions such as chronic diseases (e.g., diabetes, hypertension, cardiovascular diseases, and degenerative osteoarthritis) and mental disorders are prevalent among older adults. Although chronic diseases can be monitored or controlled clinically, mental disorders are difficult to diagnose and are thus often overlooked. Risk and protective factors for depressive symptoms in the elderly encompass physical, psychological, and social elements, among others [2, 3]. Regarding employment status and depressive symptoms, previous reviews have demonstrated a bidirectional relationship [4]. Although relationships were identified, it is interesting to note that work roles did not offer any benefits in alleviating depressive symptoms among older individuals in a Korean study [5]. What’s more, systematic reviews of longitudinal studies have demonstrated a beneficial effect of retirement on mental health [6, 7]. However, the included studies were highly heterogeneous, revealing a possible knowledge gap. Moreover, few studies have focused on the effects of employment on the mental health of Asian populations, such as the Taiwanese population. Thus, despite the cultural differences and imbalanced demographic structure throughout Taiwan, studies are needed to understand the effects of employment status on the mental health of older adults.

Population aging, and consequently workforce aging, is occurring rapidly in Taiwan. These trends are associated with an increase in the proportion of older adults who continue working. However, few studies have been conducted to investigate the effects of employment on the health of older adults in Taiwan. Therefore, in this study, we explored the effects of employment status on older adults’ mental health in terms of several demographic, psychosocial, and occupational factors.

## Methods

### Study population

The Taiwan Longitudinal Study on Aging (TLSA) is an ongoing prospective longitudinal study involving a nationally representative sample of 4049 individuals aged ≥ 60 years. For the TLSA, a three-stage equal-probability sampling design was adopted. The TLSA was initiated in 1989 by the Health Promotion Administration, Ministry of Health and Welfare, Taiwan [8]. A new cohort of younger individuals (age: 50–66 years) were added to the TLSA in 1996. Questionnaires were administered by trained interviewers at baseline (response rate: 92%) as well as during follow-up surveys in 1996, 1999, 2003, and 2007 (response rates: 91%, 89%, 90%, and 91%, respectively) [8]. In the present study, we analyzed the TLSA data pertaining to the period 1996–2007.

### Assessment of depressive symptoms and signs

The TLSA form, which utilizes a shorter version of the Center for Epidemiological Studies of Depression (CES-D) scale consisting of 10 items, was administered at both baseline and during follow-up assessments throughout our study period [9]. The respondents were asked about the frequency at which they experienced certain situations or feelings in the past week; they rated their responses on a 4-point scale (0, *not at all*; 1, *rarely* [*one day*]; 2, *sometimes* [*2–3 days*]; and 3, *often or chronically* [≥ *4 days*]). Two items “I was happy” and “I enjoyed life” were reversely coded. The remaining eight items were as follows: “I did not feel like eating; my appetite was poor,” “I felt everything I did was an effort,” “My sleep was restless,” “I felt depressed,” “I felt lonely,” “I felt that people disliked me,” “I felt sad,” and “I could not get going.” Higher scores indicated more severe depressive symptoms. As recommended by [10], we used a cutoff score of 10 to define the presence of depressive symptoms in our participants.

### Assessment of employment status

Employment status was determined by whether respondents had a job at the time of the surveys, using a binary assessment of 'working' or 'not working.' Additionally, factors such as employment type (whether full-time or not), job roles, industry sectors, and annual household income have also been assessed in the study.

### Covariates

In the TLSA form, questions about a wide array of characteristics were included. In the present study, we adjusted our statistical models for the following covariates: age, sex, education level, marital status, smoking habit, exercise habit, comorbidities (e.g., hypertension, diabetes, heart disease, and stroke), and engagement in volunteer activities. In addition, we considered financial burden and health burden, assessed by the questions "Does your health problem cause you stress or worry?" and "Does your own financial situation cause you stress or worry?" Moreover, we have also considered homeownership and social supports. Homeownership is confirmed if the respondent owns a home either alone or jointly with family, including houses, land, or buildings. Social support is evaluated by measuring the respondent's satisfaction with the emotional or psychological support provided by family and friends. Respondents who report being satisfied or very satisfied are deemed to have good social support. Data on these potential confounders, except age and education level, were collected through face-to-face interviews during each survey wave. Information on both age and education level was derived from data provided in 1996. Age was adjusted for the time gap between waves in 1-year increments.

### Statistical analysis

Descriptive characteristics were analyzed after excluding missing and implausible extreme data. Continuous data are presented in terms of the mean and standard deviation values, and categorical data are presented in terms of the number and percentage values. The chi-square and *t* tests were used to compare categorical and continuous data, respectively. Statistical significance was indicated by a two-sided *P* value of < 0.05. Logistic regression was performed to analyze the associations between employment status and the aforementioned dependent variables. The results were obtained in terms of odds ratios (ORs) and adjusted ORs (aORs) with corresponding 95% confidence intervals (CIs). The generalized estimating equation (GEE) was used to analyze the effects of unemployment on depressive symptoms.

**Fig. 1:**
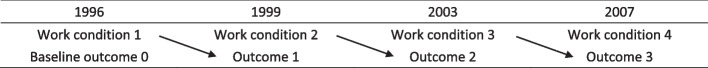
Lag 1 model. Outcomes: Center for Epidemiologic Studies Depression scale score and depression

We used a Lag 1 model (Fig. 1) with employment status and mental health outcomes as the independent and dependent variables, respectively. The covariates used in the GEE models were derived from data collected in each survey wave, which signified that the data were nonstatic, with the exception of sex and education level.

All statistical analyses were performed using JMP (version 17; SAS Institute, Cary, NC, USA) and SPSS Statistics Standard (version 25 for MacOS; IBM, New York, NY, USA).

## Results

**Fig. 2:**
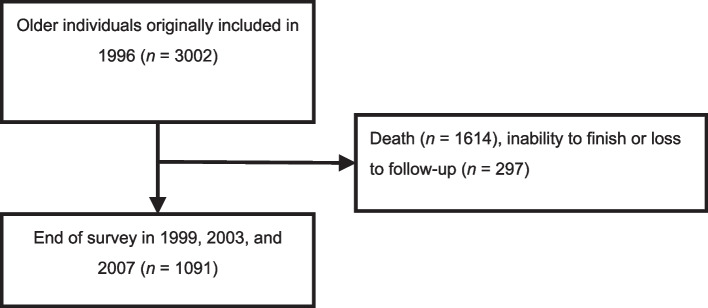
Data selection process for the longitudinal analysis

The mean CES-D scores (for all four survey waves) of employed and unemployed older adults were 3.21–4.27 and 5.39–6.49, respectively (Table 1). Mental health was better and depressive symptom prevalence was lower among employed older adults than among unemployed ones (Supplementary Fig. 1). The logistic regression analysis performed using data collected in 1996, 1999, and 2003 (Table 2 and Supplementary Fig. 2) revealed that unemployment was exerted a significant adverse effect on depressive symptoms. Next, we performed repeated measures analysis by using data from all four survey waves (the number of eligible participants in 1996 was 3002; we included 1091 individuals who had completed the surveys conducted in 1999, 2003, and 2007). The data selection process is depicted in Fig. 2. The demographic characteristics of our cohort are summarized in Supplementary Table 1. The mean age of the 1996 cohort was 72 years. In this cohort, 50.3% were men, 23% had ≥ 7 years of education, 21.5% had a smoking habit, 63.2% had an exercise habit, 64.3% were married, 32.3% had hypertension, 8.5% had diabetes, 20.1% had heart disease, 2.3% had a stroke history, 5% had a volunteer activity history, 10.9% had economic burden, and 14.1% had health burden. In 1996, 16.7% of all participants were employed; however, this percentage decreased to 2.9% in 2007.

**Table 1:** Depression-related characteristics of older individuals with different employment statuses in different years

Variables	1996		1999		2003		2007	
Mean ± SD/N (%)	Worker	No-work	Worker	No-work	Worker	No-work	Worker	No-work
	1532 (29.9%)	3599 (70.1%)	1011 (22.8%)	3429 (77.2%)	589 (15.6%)	3189 (84.4%)	1198 (26.4%)	3336 (73.6%)
Age	60.25 ± 7.44	69.45 ± 8.74	59.27 ± 7.02	68.37 ± 8.61	65.24 ± 6.57	73.27 ± 8.32	68.2 ± 5.37	75.83 ± 8.01
CES-D score	4.27 ± 5.13	6.49 ± 6.36	3.48 ± 4.45	6.07 ± 6.43	3.75 ± 4.6	5.59 ± 5.99	3.21 ± 4.26	5.39 ± 6.12
Depression	193 (13.1%)	847 (26.2%)	89 (9.0%)	748 (24.1%)	59 (10.1%)	590 (20.5%)	108 (9.1%)	625 (21.0%)

*CES-D scale* Center for Epidemiologic Studies Depression scale

The CES-D scale score ranges from 0 to 30. A CES-D score of ≥ 10 indicates depression

**Table 2:** Cross-sectional relationship between depression and unemployment in older individuals in 1996, 1999, 2003, and 2007

Variable	1996	1999	2003	2007
	aOR (95% CI)	aOR (95% CI)	aOR (95% CI)	aOR (95% CI)
Depression	1.64 (1.14—2.36)^**^	1.85 (1.39—2.46)^***^	1.73 (1.24—2.41)^**^	1.44 (1.00—2.07)

The CES-D scale score ranges from 0 to 30. A CES-D scale score of ≥ 10 indicates depression

*CES-D scale* Center for Epidemiologic Studies Depression scale, *CI* confidence interval, *OR* odds ratio, *aOR* adjusted odds ratio

The models were adjusted for various factors, including age, sex, education level, marital status, smoking habit, exercise habit, comorbidities (e.g., hypertension, diabetes, heart disease, and stroke), engagement in volunteer activities, economic burden, and health burden in 1996, 1999, 2003, and 2007

^*^P < 0.05, **P < 0.01, and ***P < 0.001; chi-square test

Next, we used GEE models to investigate the effects of unemployment on depressive symptoms. As shown in Table 3, being employed exerted a significant, protective effect on depressive symptoms, as noted in the subsequent surveys conducted after 3 to 4 years (aOR [95% CI] = 0.679 [0.465–0.989]). Notably, no significant differences were noted between employed and unemployed older adults in marital status, smoking habit, hypertension and diabetes history, or stroke history. The dependent variables were found to be strongly correlated with the participants’ baseline mental status, health burden, and social supports.

**Table 3:** Correlations between different variables and depressive CES-D scale scores

Model^1^ for CES-D score (continuous data)				
Variables	B	95% CI		*p*-value
Gender (male vs. female)	0.897	0.333	1.462	0.002
Education level (< high school vs. ≥ high school)	0.769	0.248	1.290	0.004
Age	0.042	-0.008	0.093	0.099
Marital status (married and living together vs. others)	-0.212	-0.733	0.310	0.426
Smoking habit (yes vs. no)	0.349	-0.228	0.926	0.236
Exercise habit (yes vs. no)	-0.827	-1.326	-0.329	0.001
Hypertension or Diabetes (yes vs. no)	0.386	-0.086	0.857	0.109
Heart disease (yes vs. no)	0.687	0.090	1.283	0.024
Stroke (yes vs. no)	1.198	-0.276	2.671	0.111
Volunteer activity (yes vs. no)	-0.892	-1.538	-0.246	0.007
Economic burden (yes vs. no)	1.577	0.877	2.277	< .001
Health burden (yes vs. no)	2.050	1.490	2.611	< .001
Homeownership (yes vs. no)	-0.567	-1.174	0.040	0.067
Good social supports	-1.304	-1.904	-0.703	< .001
Baseline depressive symptoms	2.442	1.703	3.180	< .001
**Employment status (working vs. not working)**	**-0.654**	**-1.262**	**-0.045**	**0.035**
Model^2^ for depression (dichotomous data)				
Variables	aOR	95% CI		*p*-value
Gender (male vs. female)	1.305	1.000	1.703	0.050
Education level (< high school vs. ≥ high school)	1.415	1.034	1.935	0.030
Age	1.013	0.989	1.038	0.282
Marital status (married and living together vs. others)	0.961	0.766	1.207	0.733
Smoking habit (yes vs. no)	1.076	0.808	1.433	0.615
Exercise habit (yes vs. no)	0.861	0.692	1.072	0.181
Hypertension or Diabetes (yes vs. no)	0.995	0.795	1.246	0.965
Heart disease (yes vs. no)	1.324	1.035	1.695	0.026
Stroke (yes vs. no)	1.526	0.908	2.565	0.110
Volunteer activity (yes vs. no)	0.454	0.248	0.832	0.011
Economic burden (yes vs. no)	1.413	1.084	1.842	0.011
Health burden (yes vs. no)	2.152	1.723	2.688	< .001
Homeownership (yes vs. no)	0.823	0.631	1.072	0.148
Good social supports	0.603	0.475	0.766	< .001
Baseline depressive symptoms	2.087	1.634	2.664	< .001
**Employment status (working vs. not working)**	**0.679**	**0.465**	**0.989**	**0.044**

The correlations were identified through a repeated measures analysis (generalized estimating equation model)

Models 1 and 2 were adjusted for various factors, including age, sex, education level, marital status, smoking habit, exercise habit, comorbidities (e.g., hypertension, diabetes, heart disease, and stroke), engagement in volunteer activities, economic burden, health burden, homeownership, social supports, baseline depressive symptoms, and working conditions 3 to 4 years ago. These variables were derived from data collected in each survey wave, with the exception of gender and education level, which were based on the information collected in 1996

A CES-D scale score of ≥ 10 indicates depressive symptoms and depression

*CES-D scale* Center for Epidemiologic Studies Depression scale, *CI* confidence interval; *OR* odds ratio, *aOR* adjusted odds ratio

## Discussion

In this study, we used data from a nationwide representative survey to identify the correlation between employment status and mental health among older adults in Taiwan. Robust findings were obtained through a repeated measures analysis: employment was found to reduce the risk of developing depressive symptoms within 3 to 4 years by 32%.

Our results revealed that participants with a low education level and those with economic or health burden were more likely to have depressive symptoms than were the others; these findings corroborate those of [11, 12]. We further noted that older adults participating in volunteer activities experienced improved mental health, consistent with the findings of [13]. Studies on employment status in older adults have mostly adopted a cross-sectional design. For instance, studies conducted in Turkey, Bangladesh, and Japan have reported that unemployment is a crucial risk factor for depression in older adults [14–16]. In the present study, we confirmed the longitudinal relationship between employment status and depressive symptoms among Taiwanese older adults.

Retirement, the 10th most stressful life transition event, results in social and psychological changes, which may exert positive or negative effects on mental health [17]. In a Finnish study, the observed trends in antidepressant medication purchase indicated that retirement is beneficial for mental health [18]. However, in Japan, retirement was noted to increase the risk of depressive symptoms; this effect was relatively significant among men with a lower occupational class than among the others [19]. A review study revealed that unlike Asian studies, Western studies have generally reported the positive effects of retirement on mental health [20]. In a systemic review, all studies, except four Asian studies, were found to indicate that retirement reduces depression risk [7]. In the present study, we noted a similar trend: retirement (i.e., unemployment) led to the deterioration of mental health in Taiwanese older adults. While socio-cultural differences between countries may not be readily adjustable in the short term, clarifying welfare systems closely related to retirement, categorizing occupations and industries, expanding part-time and other non-traditional forms of employment, and exploring emerging work environments such as international cooperation are all valuable research avenues. These areas merit further investigation to understand their impact on the health of older workers. Findings from such studies will be critical in formulating recommendations for future policy enhancements.

A major strength of our study is the inclusion of a nationally representative sample of community-dwelling older adults in Taiwan. After adjustments for potential confounders, our methodology was sufficiently powerful to reveal meaningful associations. A longitudinal study would provide further insights into the relative role of age and age-related differences in mediating the effects of employment status on mental health as well as relevant cohort effects. Moreover, although we used a large, multiwave design rather than a cross-sectional or single follow-up design, the sensitivity and specificity of the Chinese versions of CES-D have been validated [10]. Therefore, the methods used in this study to collect data from Taiwan’s older population were reliable.

The present study has several limitations. The first limitation was the loss of follow-up data because of death or loss to follow-up. Therefore, the attrition effect could not be eliminated. Moreover, most deceased participants were men, less educated, and smokers [21]. Therefore, the sample of participants with depressive symptoms may be underrepresented. Thus, the observed relationship may be similar to the null hypothesis. Second, all variables were self-reported and thus were subject to recall bias. Nevertheless, some parts of the questionnaire were validated by our well-trained interviewers; they also performed spot checks to ensure that all responses were collected accurately. The dependent variables were dependent on the subsequent surveys, which minimized recall bias concerning the target diseases or symptoms. However, each participant might have had a different definition of work. The differences in having full-time, part-time, contractual, and no work might have influenced the interpretation of employment status reported in the TLSA. In addition, the factors influencing the progression of depressive symptoms are complicated. For instance, if participants experienced changes in their employment status within the three- to four-year period or shortly before the subsequent survey wave, this dynamic could attenuated the observed association between employment status and later depressive symptoms. However, we believe that the multi-wave nature of our study helps mitigate this effect to some extent. As such, we might not have adjusted for all potential factors; nevertheless, our results corroborate those of other studies.

## Conclusion

Our results indicate that unemployment among community-dwelling older adults in Taiwan increases the risk of depressive symptoms both cross-sectionally and longitudinally. The findings may facilitate the development of tailored programs aimed at enhancing the well-being of older adults. However, the dynamic relationship of mental health with relevant factors should be investigated in future studies. Moreover, studies must be conducted to analyze the negative effects of employment status on the mental health of these individuals and to elucidate the underlying mechanisms.

Although our study has identified the impact of employment status on mental health, recommending whether older adults should continue working remains challenging. This complexity stems from generational differences within our study population, along with evolving social, cultural, and socio-environmental factors that introduce diverse work styles and increased flexibility, thus broadening personal choice. Nevertheless, we believe that when individuals are physically capable, engaging in work that does not negatively impact their health can be advantageous both for the individual and for society as a whole.

### Supplementary Information

Supplementary Material 1.

## Data Availability

The study data can be accessed through application to the Health and Welfare Data Science Center, Ministry of Health and Welfare, Taiwan (https://dep.mohw.gov.tw/DOS/cp-5283-63826-113.html).
