# Supplementary material for: Employment status and depressive symptoms in taiwanese older adults: an 11-year prospective cohort study
Source: BMC Geriatr. 2024 Aug 9;24:671. doi: 10.1186/s12877-024-05258-w (PMC11312394; doi:10.1186/s12877-024-05258-w)
Supplement: Supplementary file 1 — Supplementary Material 1. [file 12877_2024_5258_MOESM1_ESM.docx]

**Supplementary Figure 1.**

CES-D scores (A) and depression prevalence (B) in working and nonworking older individuals in 1996, 1999, 2003, and 2007.

CES-D scale, Center for Epidemiologic Studies Depression scale. A CES-D score of ≥10 indicates depression.

*

**Supplementary Figure 2.**

Correlation of unemployment with depression.

A Center for Epidemiologic Studies Depression score of ≥10 indicates depression disorder.

The models were adjusted for various factors, including age, sex, education level, marital status, smoking habit, exercise habit, comorbidities (e.g., hypertension, diabetes, heart disease, and stroke), engagement in volunteer activities, economic burden, and health burden in 1996, 1999, 2003, and 2007. CI, confidence interval; OR, odds ratio; aOR, adjusted odds ratio. *Nonsignificant.

**Supplementary Table 1.** Demographic characteristics of the participants finally included in the longitudinal analysis (*n* = 1091).

| Variables: N (%) or mean ± SD | 1996 | 1999 | 2003 | 2007 |
| --- | --- | --- | --- | --- |
| Age | 72.39 ± 4.02 | 75.39 ± 4.02 | 79.39 ± 4.02 | 83.39 ± 4.02 |
| Male gender | 549 (50.3%) | 549 (50.3%) | 549 (50.3%) | 549 (50.3%) |
| Education level: equal to or above High school (≥ 7years) | 250 (22.9%) | 250 (22.9%) | 250 (22.9%) | 250 (22.9%) |
| Marital status (Married and living together) | 701 (64.3%) | 640 (58.7%) | 544 (49.9%) | 458 (42.0%) |
| Smoking habit | 235 (21.5%) | 210 (19.2%) | 161 (14.8%) | 118 (10.8%) |
| Exercise habit | 690 (63.2%) | 742 (68.0%) | 725 (66.5%) | 567 (52.0%) |
| Hypertension | 352 (32.3%) | 375 (34.4%) | 480 (44.0%) | 517 (47.4%) |
| Diabetes | 93 (8.5%) | 126 (11.5%) | 159 (14.6%) | 179 (16.4%) |
| Heart diseases | 219 (20.1%) | 217 (19.9%) | 275 (25.2%) | 290 (26.6%) |
| Stroke | 25 (2.3%) | 48 (4.4%) | 79 (7.2%) | 119 (10.9%) |
| Volunteer activity | 54 (5.0%) | 67 (6.1%) | 48 (4.4%) | 37 (3.4%) |
| Economic burden | 119 (10.9%) | 275 (25.2%) | 225 (20.6%) | 184 (16.9%) |
| Health burden | 154 (14.1%) | 383 (35.1%) | 356 (32.6%) | 324 (29.7%) |
| Homeownership | 943 (86.4%) | 919 (84.2%) | 900 (82.5%) | 911 (83.5%) |
| Good social supports | 811 (74.3%) | 833 (76.4%) | 827 (75.8%) | 746 (83.1%) |
| Annual household income < 60 USD* | 156 (95.7%) | 171 (31.8%) | 177 (34.0%) | 481 (44.1%) |
| Working status | 182 (16.7%) | 117 (10.7%) | 62 (5.7%) | 32 (2.9%) |
| CES-D score | 5.44 ± 5.74 | 5.26 ± 5.64 | 5.55 ± 5.98 | 6.02 ± 6.31 |
| Depressive symptoms | 214 (20.6%) | 209 (20.0%) | 186 (18.6%) | 223 (25.1%) |

Data regarding all variables, except age and education level, were collected through face-to-face interviews during each survey wave. * The annual household income has too many missing data points, and the percentage reported is based on the percentage of available data.
